# Association of *AXIN1 rs12921862 C/A* and *rs1805105 G/A* and *CTSB rs12898 G/A* polymorphisms with papillary thyroid carcinoma: A case–control study

**DOI:** 10.1002/jcla.24804

**Published:** 2022-12-12

**Authors:** Shaghayegh Saljooghi, Zahra Heidari, Mohsen Saravani, Mahnaz Rezaei, Saeedeh Salimi

**Affiliations:** ^1^ Department of Clinical Biochemistry, School of Medicine Zahedan University of Medical Sciences Zahedan Iran; ^2^ Department of Internal Medicine Zahedan University of Medical Sciences Zahedan Iran; ^3^ Cellular and Molecular Research Center Resistant Tuberculosis Institute, Zahedan University of Medical Sciences Zahedan Iran; ^4^ Department of Clinical Biochemistry, School of Medicine Shahid Beheshti University of Medical Sciences Tehran Iran

**Keywords:** *AXIN1*, *CTSB*, papillary thyroid cancer, polymorphism, tumor size

## Abstract

**Background:**

Papillary thyroid cancer (PTC) is the most common type of thyroid cancer which its precise etiology remains unknown. However, environmental and genetic factors contribute to the etiology of PTC. Axis inhibition protein 1 (Axin1) is a scaffold protein that exerts its role as a tumor suppressor. In addition, Cathepsin B (Ctsb) is a cysteine protease with higher expression in several types of tumors. Therefore, the aim of this study was to investigate the possible association of *AXIN1* rs12921862 C/A and rs1805105 G/A and *CTSB* rs12898 G/A polymorphisms with PTC susceptibility.

**Materials & Methods:**

In total, 156 PTC patients and 158 sex‐, age‐, and BMI‐matched control subjects were enrolled in the study. *AXIN1* rs12921862 C/A and rs1805105 G/A and *CTSB* rs12898 G/A polymorphisms were genotyped using the PCR–RFLP method.

**Results:**

There was a relationship between *AXIN1* rs12921862 C/A polymorphism and an increased risk of PTC in all genetic models except the overdominant model. The *AXIN1* rs1805105 G/A polymorphism was associated with an increased PTC risk only in codominant and overdominant models. The frequency of *AXIN1 A*_*rs12921862*_ *A*_*rs1805105*_ haplotype was higher in the PTC group and also this haplotype was associated with an increased risk of PTC. Moreover, the *AXIN1* rs12921862 C/A polymorphism was not associated with PTC clinical and pathological findings, but *AXIN1* rs1805105 G/A polymorphism was associated with almost three folds of larger tumor size (≥1 cm). There was no association between *CTSB* rs12898 G/A polymorphism and PTC and its findings.

**Conclusion:**

The *AXIN1* rs12921862 C/A and rs1805105 G/A polymorphisms were associated with PTC. *AXIN1 rs1805105 G/A* polymorphism was associated with higher tumor size.

## Graphical abstract

Papillary thyroid cancer (PTC) is the most common type of thyroid cancer which genetic and environmental factors play key roles in its pathogenesis. Axin1 is a scaffold protein which plays a tumor suppressor role. Cathepsin B is a cysteine protease enzyme with higher expression in tumors. Regarding the role of Axin1 and Cathepsin B on tumorigenesis, the aim of this study was to evaluate the effect of Axin1 and Cathepsin B polymorphisms on PTC. The *AXIN1 rs12921862* and *rs1805105* polymorphisms was associated with increased risk of PTC. The *AXIN1* rs1805105 polymorphism was associated with larger tumor size (≥1 cm). There was no association between *CTSB* rs12898 polymorphism and PTC.

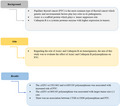

## 1 INTRODUCTION

Thyroid cancer is the most common tumor in the endocrine system, accounting for about 1% of all cancer cases to date.^1^ Different types of thyroid cancer have been known, such as follicular thyroid cancer (FTC), anaplastic thyroid cancer (ATC), and medullary thyroid cancer (MTC). The most prevalent form of thyroid cancer is papillary thyroid cancer (PTC), accounting for almost 80% of all cases.^2^, ^3^ PTC is a well‐differentiated thyroid carcinoma initiated from follicular cells that present a good prognosis when adequately treated. About 95% of patients who suffer from PTC can be cured by surgery and accessory therapies and have a 10‐year survival.^4^ On the other hand, some patients show aggressive tumor behavior and suffer from recurrence and death even after regular treatment. Lymph node metastasis (LNM) is a crucial factor that aggravates the risk of recurrence and mortality in patients suffering from PTC.^5^ It seems that the PTC is more frequent in females compared to males by three times.^6^

Despite numerous studies, the precise etiology of PTC remains unknown. Environmental factors, such as radiation exposure, hormonal factors, family history, and genetic factors contribute to the etiology of PTC.^7^ Several evidences of family studies revealed the role of genetic factors in the etiology of PTC. In addition, genetic factors appear to be more pivotal in the origin of papillary carcinoma than follicular carcinomas. Furthermore, there is evidence that shows some ethnicities may be more susceptible to the effects of ionizing radiation. A wide range of genetic alterations, including chromosomal rearrangements and point mutations, have been implicated in the development of PTC. Chromosomal rearrangements are likely to link to radiation exposure, whereas the origin of point mutation remains unknown.^8^ Studies have confirmed that mutations in some genes, such as microRNA let‐7a‐2, mammalian target of rapamycin (mTOR) and RAC(Rho family)‐alpha serine/threonine‐protein kinase(AKT1), Mouse double minute 2 homolog(MDM2), Forkhead box protein O1 (FOXO1), Fas receptor genes are likely to associate with the risk of PTC.^10^, ^11^

Recently, several evidences imply the effect of the Wnt (Wingless/Integrated) signaling pathway in the etiology of PTC. Normal thyroid cells could express several proteins, including Frizzled receptors (Fzd), Disheveled (Dvl), and Wnt proteins. Moreover, TSH‐dependent overexpression of Wnt1 and Glycogen synthase kinase‐3 beta(GSK3β) inhibition in relation to thyroid cell proliferation has been shown.^12^ In addition, genetic variations in some proteins involved in the Wnt pathway, such as APC, β‐catenin, and Axin, have been indicated in some types of thyroid carcinomas.^12^, ^13^ In a study performed by Kurihara et al,^14^ *AXIN1* mutations was observed in 81.8% of anaplastic thyroid cancers (ATCs) patients, resulting in amino acid substitution. The different mutations found in the *AXIN1* gene affect the domains for interaction with Adenomatous polyposis coli (APC), β‐catenin, and Dvl, and the G‐protein regulatory domain, and thus influence the role of Axin1 as a negative regulator of β‐catenin in PTC.^14^

Axis inhibition protein 1 (Axin1) is a multi‐domain scaffold protein that affects the β‐catenin levels and localization when the Wnt pathway activates. This protein contributes to the promotion and improvement of several complications, such as Caudal duplication anomalies, atrial septal defect, non‐small‐cell lung cancer (NSCLC), breast cancer, gastrointestinal cancer, and colorectal cancer.^15^ *AXIN1* gene lies on chromosome 16p13.3 (65‐kb), and also its protein product plays a critical role in the embryonic development.^16^ The interaction of Axin1 protein with several proteins acts as a leading negative regulator in the Wnt signaling pathway. In recent years, in silico analysis suggested that overexpression of the CDH16 gene, whose expression act as a biomarker for thyroid cancer, negatively regulates the *AXIN1* expression in thyroid tissue.^17^ There are several genetic variants in the *AXIN1* gene which their effects on cancer have been reported.^18^

Cathepsin B (CTSB) is a cysteine protease enzyme whose role in the degradation of lysosomal proteins has been well known. CTSB is a tumor biomarker because it promotes tumor progression, and also its expression is elevated in many types of tumors. Indeed, evidence showed higher expression of *CTSB* in several types of solid tumors. Overexpression of *CTSB* has been seen in PTC cells, associated with lymph node metastasis and advanced N stage.^19^ The *CTSB* gene is located on chromosome 8(8p23.1). There are several genetic polymorphisms in the *CTSB* gene whose effects on several cancers have been studied.^20^, ^21^

Given the effects of AXIN1 and CTSB in tumorigenesis and lack of published study on the effects of their polymorphisms on thyroid cancer; therefore, for the first time, this study was conducted to investigate the association between *AXIN1* rs12921862 C/A and rs1805105 G/A and *CTSB* rs12898 G/A polymorphisms and the risk of PTC in Iranian population.

## 2 MATERIALS AND METHODS

### 2.1 Study group

A case–control study was conducted on 156 PTC patients and 158 sex‐, age‐, and BMI‐ matched controls who were referred to the outpatient endocrinology clinic of the Zahedan (southeast of Iran).

The detection of PTC was evaluated by the pathological findings of ultrasonography‐guided fine needle aspiration biopsy or resected specimens according to the revised American Thyroid Association Management guidelines.^22^ Two pathologists were employed to confirm all samples. The exclusion criteria for the study included the patients having the history of thyroid disease or thyroid surgery, those who has had neck irradiation, iodinated contrast material exposure within the last 6 months, and other types of cancer.

The control group had no previous history of cancer, endocrine disorders, diabetes mellitus, renal or hepatic dysfunction, significant neurological or psychological illness.

All cases and controls were selected between January 2017 and February 2019. The study protocol was approved by the ethical committee of Zahedan University of Medical Sciences, and also the participants signed a written informed consent form.

### 2.2 DNA extraction

Of the 500 μl whole K2 EDTA‐treated peripheral blood, the human genomic DNA was isolated through the salting‐out method and kept at −20°C in nuclease‐free distilled water. Gel electrophoresis using 1% agarose gel and NanoDrop spectrophotometer were used to measure the quality and quantity of the extracted DNA, respectively.

### 2.3 Genotyping

Genotyping of *AXIN1* rs12921862 C/A, rs1805105 G/A, and *CTSB* rs12898 G/A variants was done by restriction fragment length polymorphism‐polymerase chain reaction (RFLP–PCR) method as previously described (Table 1).^18^, ^20^ For all polymorphisms, the reaction tubes containing 9 μl of 2X master mix, 1 μl each primer (10 μM), 6 μl deionized water and, 100 ng DNA template were added to a 20μl PCR reaction tube. PCR protocol was performed in VeritiTM 96‐well Thermal Cycler ABI instrument with a pre‐denaturation step at 95°C for 5 min, followed by 30 cycles of denaturation at 95°C for 40 s, annealing at a particular temperature based on Table 1, for 35 s, extension at 72°C for the 30 s, and the final extension at 72°C for 5 min. In the next step, 3.5 μl of PCR product of *AXIN1* rs12921862 C/A, rs1805105 G/A and, *CTSB* rs12898 G/A polymorphisms was incubated by 10 IU of ScrF1(ER1421, Thermo Fisher Scientific), FokI (FD2144, Thermo Fisher Scientific) and, Hind III(FD0504, Thermo Fisher Scientific) restriction enzymes at 37°C overnight. The digested fragments were electrophoresed on 3% agarose gel stained with safe stain.

**TABLE 1:** The primer sequences and PCR‐RFLP conditions for genotyping of *AXIN1* and *CTSB* polymorphisms

Polymorphism	Primer sequence (5′–3′)	Annealing Temperature (°C)	Restriction Enzyme	Fragments (bp)
*AXIN1* rs12921862	F‐CTCACGCCAGTGCCTCTACT R‐ATGCCATCCATGTGGAAACT	55	ScrFI	C allele:216 A allele:110 + 106
*AXIN1* rs1805105	F‐CTGGATACCTGCCGACCTTA R‐ACCTTTCCCTGGCTTGTTCT	54	FokI	C allele:245 T allele:186 + 59
*CTSB* rs12898	F‐GAGGATTCAGCTCATAAAACAAG R‐CAAACCAGTGGCATACAAATTCA	56	Hind III	A allele: 160 G allele: 139 + 21

### 2.4 Statistical analysis

Statistical analysis was performed using SPSS version 23 (IBM SPSS Statistics for Windows, Version 23.0). The clinical and demographic specifications were evaluated using Fisher exact test or an independent Student's *t*‐test.

Differences between the genotypic and allelic distribution of the study groups, and clinical features of PTC were calculated using SNPStats (http://bioinfo.iconcologia.net/snpstats/start.htm) in various genetic models by obtaining the odds ratio (OR) and their CI of 95% (95% CI). Also Linkage disequilibrium was estimated using SNPStats. Hardy Weinberg equilibrium was calculated with Chi‐square test.

## 3 RESULTS

The demographic and clinical findings of PTC and control groups are presented in Table 2.

**TABLE 2:** Demographic and clinical characteristics of papillary thyroid carcinoma patients and controls

	PTC (*n* = 156)	Control (*n* = 158)	*p*‐value
Age	36.1 ± 12.1	33.8 ± 10.4	0.80
Sex			
Male	29 (18.6)	28 (18)	
Female	127 (81.4)	106 (82)	0.77
Location			
Right lobe	69 (44.2)		
Left lobe	70 (44.9)		
Both lobes	17 (10.9)		
Tumor size			
<1 cm	30 (19.2)		
≥1 cm	111 (71.2)		
Unknown	15 (9.6)		
TNM stage			
I	88 (56.4)		
II	18 (11.5)		
III	16 (10.3)		
IV	16 (10.3)		
Unknown	18 (11.5)		
N stage			
N0	91 (58.3)		
N1	46 (29.5)		
Unknown	19 (12.2)		
M stage			
M0	132 (84.6)		
M1	5 (3.8)		
Unknown	19 (12.2)		
Vascular invasion			
Positive	20 (12.8)		
Negative	118 (75.6)		
Unknown	18 (11.5)		
Capsular invasion			
Positive	22 (14.1)		
Negative	116 (74.4)		
Unknown	18 (11.5)		
Extrathyroidal expansion			
Positive	17 (10.9)		
Negative	120 (76.9)		
Unknown	19 (12.2)		

*Note*: The quantitative variables are presented as mean ± SD and are analyzed by Student *t*‐Test.

The qualitative variables are analyzed by Fisher exact Test.

The *p*‐value < 0.05 was considered to be statistically significant.

### 3.1 AXIN1 and CTSB polymorphisms and PTC susceptibility

**FIGURE 1:**
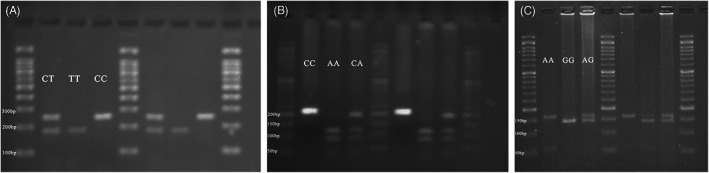
Agarose gel electrophoresis images of polymerase chain reaction restriction fragments length polymorphism (PCR–RFLP) for (A) *AXIN1* rs1805105, (B) *AXIN1* rs12921862, and (C) *CTSB* rs12898 polymorphisms.

The results of agarose gel electrophoresis for *AXIN1* rs1805105 (Figure 1A), *AXIN1* rs12921862 (Figure 1B) and *CTSB* rs12898 (Figure 1C) polymorphisms using the PCR–RFLP method are shown in Figure 1.

The *AXIN1* rs12921862, rs1805105, and *CTSB* rs12898 polymorphisms were not deviated from the Hardy–Weinberg equilibrium (*p* = 1, *p* = 0.45 and *p* = 0.09, respectively). The *AXIN1* rs12921862 CA and AA genotypes were more frequent in PTC group, and could increase the PTC risk in codominant model (OR = 2.77, 95% CI = 1.21–6.36 and OR = 4.69, CI = 2.07–10.61, *p* = 2e‐04, respectively). Moreover, rs12921862 C/A variant was associated with 3.72, 2.08‐, 1.97, and 2.06fold increased PTC risk in dominant (OR = 3.72, CI = 1.69–8.19, *p* = 5e‐04), recessive (OR = 2.08, CI = 1.34–3.24, *p* = 0.001), log‐additive (OR = 1.97, CI = 1.40–2.76, *p* = 1e‐04) and allelic (OR = 2.06, CI = 1.46–2.89, *p* = 5e‐04) models, respectively (Table 3). The frequency of rs1805105 GA genotype was higher in PTC patients and also rs1805105 G/A polymorphism was associated with 1.73 and 1.78‐fold increased PTC risk only in codominant (OR = 1.73, CI = 1.07–2.77, *p* = 0.03) and overdominant (OR = 1.78, CI = 1.11–2.84, *p* = 0.016) models (Table 3). The analysis of the association between AXIN1 polymorphisms and PTC after adjustment for age, sex and BMI, confirmed the above findings.

**TABLE 3:** Allelic and genotypic frequency of *AXIN1* polymorphisms in PTC patients and control group

	Control (*N* = 169)	PTC (*N* = 156)	*p*‐value	OR (95% CI)
*rs12921862*				
CC, *n* (%)	100 (59)	64 (41)		1
CA, *n* (%)	60 (36)	65 (42)		
AA, *n* (%)	9 (5)	27 (17)		
Codominant			**2e‐04**	2.77 (1.21–6.36)
				4.69 (2.07–10.61)
Dominant (CA + AA vs. CC)			**5e‐04**	3.72 (1.69–8.19)
Recessive (AA vs. CA + CC)			**0.001**	2.08 (1.34–3.24)
Overdominant (CC + AA vs. CA)			0.250	0.77 (0.49–1.21)
Log‐additive (AA vs. CA vs. CC)			**1e‐04**	1.97 (1.40–2.76)
Allele				
C, *n* (%)	260 (77)	193 (62)		1
A, *n* (%)	78 (23)	119 (38)	**5e‐04**	2.06 (1.46–2.89)
*rs1805105*				
GG, *n* (%)	119 (70)	94 (60)		
GA, *n* (%)	44 (26)	60 (43)		
AA, *n* (%)	6 (4)	2 (2)		
Codominant			**0.030**	1.73 (1.07–2.77)
				0.42 (0.08–2.14)
Dominant (GA + AA vs. GG)			0.054	1.57 (0.99–2.49)
Recessive (AA vs. GG + GA)			0.180	0.35 (0.07–1.77)
Overdominant (GG + AA vs. GA)			**0.016**	1.78 (1.11–2.84)
Log‐additive (AA vs. GA vs. GG)			0.181	1.32 (0.88–2.00)
Allele				
G, *n* (%)	282 (83)	248 (77)		—
A, *n* (%)	56 (17)	64 (23)	0.225	1.30 (0.87–1.93)

*Note*: Logistic regression analysis was used to calculate the independent effect of each polymorphism on PTC risk after adjusting for age, sex, and BMI.

Bold values are statistically significant (*p* < 0.05).

Haplotype evaluation of *AXIN1* rs12921862 C/A and rs1805105 G/A polymorphisms indicated, higher *A*_*rs12921862 C/A*_ *T*_*rs1805105*_ haplotype in PTC group (Table 4) and this haplotype was associated with 12.34‐ fold incresead risk of PTC (OR = 12.34, CI = 2.98–51.14, *p* = 6e‐04). The linkage disequilibrium was weak between *AXIN1* rs12921862, rs1805105 polymorphisms (*D*′ = 0.09, *r* = ‐0.03).

**TABLE 4:** Haplotypes frequency of *AXIN1* rs12921862 and rs1805105 polymorphisms in PTC patients and controls

*rs12921862*/*rs1805105*	Case	Control	*p*‐value	OR (95% CI)
CG	0.512	0.614	—	1
AG	0.284	0.220	0.084	1.42 (0.95–2.11)
CA	0.107	0.155	0.42	0.79 (0.45–1.40)
AA	0.097	0.011	**6e‐04**	12.34 (2.98–51.14)

*Note*: Logistic regression analysis was used to calculate the independent effect of each haplotype onPTC risk.

Bold values are statistically significant (*p* < 0.05).

The frequency of *CTSB* rs12898 G/A genotype did not differ between PTC and control subjects, and also this variant was not associated with PTC in any genetic model (Table 5). After adjustment for age, sex and BMI the findings were confirmed.

**TABLE 5:** Allelic and genotypic frequency of *CTSB rs12898 G/A* polymorphisms in PTC patients and control group

	Control (*N* = 169)	PTC (*N* = 127)	*p*‐value	OR (95% CI)
*rs12898*				
GG, *n* (%)	76 (45)	64 (41)		1
GA, *n* (%)	67 (40)	62 (40)		
AA, *n* (%)	26 (15)	30 (19)		
Codominant			0.611	1.10 (0.68–1.77)
				1.37 (0.74–2.55)
Dominant (GA + AA vs. GG)			0.472	1.17 (0.76–1.82)
Recessive (AA vs. GA + GG)			0.360	1.31 (0.74–2.33)
Overdominant (GG + AA vs. GA)			0.992	1.00 (0.64–1.57)
Log‐additive (AA vs. GA vs. GG)			0.341	1.16 (0.86–1.56)
Allele				
G *n* (%)	219 (65)	190 (61)		1
A, *n* (%)	119 (35)	122 (39)	0.329	1.18 (0.86–1.62)

*Note*: Logistic regression analysis was used to calculate the independent effect of each polymorphism onPE risk after adjusting for age, sex, and BMI.

*p* < 0.05 was considered statistically significant.

### 3.2 AXIN1 and CTSB polymorphisms and PTC findings

There was no significant association between *AXIN1* rs12921862 C/A polymorphism and clinical findings of PTC (Table 6).

**TABLE 6:** Association of *AXIN1* rs12921862 polymorphism with clinical characteristics of papillary thyroid carcinoma

Characteristics	CC	CA	AA	*p*‐value/OR (95% CI)					
				Codominant1	Codominant2	Dominant	Recessive	Overdominant	Log‐additive
Age, years									
<40	43 (42)	40 (39)	19 (19)	0.67		0.69	0.55	0.39	0.96
≥40	21 (39)	25 (46)	8 (15)	1.28 (0.62–2.64)	0.86 (0.32–2.29)	1.15 (0.58–2.25)	0.76 (0.31–1.87)	1.34 (0.69–2.60)	0.99 (0.63–1.56)
Sex									
Female	53 (42)	54 (43)	20 (16)	0.58		0.71	0.30	0.65	0.42
Male	11 (38)	11 (38)	7 (24)	0.98 (0.44–2.44)	1.68 (0.57–5.00)	1.18 (0.51–2.70)	1.70 (0.64–4.54)	0.83 (0.36–1.89)	1.25 (0.73–2.17)
Tumor size									
<1 cm	11 (37)	13 (43)	6 (20)	0.84		0.57	0.72	0.78	0.57
≥1 cm	47 (42)	45 (41)	19 (17)	0.81 (0.33–1.99)	0.74 (0.24–2.29)	0.79 (0.34–1.81)	0.83 (0.30–2.30)	0.89 (0.39–2.02)	0.85 (0.49–1.47)
N stage									
N0	37 (41)	38 (42)	16 (17)	0.99		0.94	0.98	0.96	0.95
N1	19 (41)	19 (41)	8 (18)	0.97 (0.45–2.13)	0.97 (0.35–2.68)	0.97 (0.47–2.00)	0.99 (0.39–2.51)	0.98 (0.48–2.02)	0.98 (0.60–1.60)
TNM stage									
I–II	43 (41)	47 (44)	16 (15)	0.52		0.75	0.38	0.31	0.80
III–IV	14 (44)	11 (34)	7 (22)	0.72 (0.29–1.75)	1.34 (0.46–3.93)	0.88 (0.39–1.95)	1.57 (0.58–4.25)	0.66 (0.29–1.50)	1.07 (0.62–1.85)
Extrathyroidal expansion									
Negative	48 (40)	49 (40.8)	23 (19.2)	0.55		0.31	0.44	0.66	0.28
Positive	9 (52.9)	6 (35.3)	2 (11.8)	0.65 (0.22–1.98)	0.46 (0.09–2.32)	0.59 (0.21–1.64)	0.56 (0.12–2.63)	0.79 (0.27–2.28)	0.67 (0.32–1.40)
Vascular invasion									
Negative	49 (41.5)	48 (40.7)	21 (17.8)	0.75		0.89	0.46	0.63	0.63
Positive	8 (40)	7 (35)	5 (25)	0.89 (0.30–2.66)	1.46 (0.43–4.98)	1.07 (0.41–2.80)	1.54 (0.50–4.70)	0.79 (0.29–2.11)	1.17 (0.62–2.20)
Capsular invasion									
Negative	52 (44.8)	46 (39.7)	18 (15.5)	*0.48*		0.25	0.42	0.61	0.24
Positive	7 (31.8)	10 (45.5)	5 (22.7)	1.61 (0.57–4.59)	2.06 (0.58–7.32)	1.74 (0.66–4.59)	1.60 (0.52–4.89)	1.27 (0.51–3.18)	1.45 (0.79–2.69)

*Note*: Logistic regression analysis was used for statistical analysis.

*p* <0.05 was considered statistically significant.

The frequency of *AXIN1* rs1805105 C/A polymorphism was higher in females and PTC patients with extrathyroidal expansion, but was not statistically significant (Table 7). However, this polymorphism was associated with higher tumor size (≥1 cm) in codominant (OR = 2.92, CI = 1.10–7.72, *p* = 0.043), dominant (OR = 3.05, CI = 1.16–8.04, *p* = 0.016), overdominant (OR = 2.83, CI = 1.07–7.48, *p* = 0.025) and log additive (OR = 3.03, CI = 1.17–7.84, *p* = 0.013).

**TABLE 7:** Association of *AXIN1* rs1805105 polymorphism with clinical characteristics of papillary thyroid carcinoma.

Characteristics	GG	GA	AA	*p*‐value/OR (95% CI)					
				Codominant1	Codominant2	Dominant	Recessive	Overdominant	Log‐additive
Age, years									
<40	59 (57.8)	40 (39.2)	3 (2.9)	0.25		0.53	0.11	0.79	0.36
≥40	34 (63)	20 (37)	0 (0)	0.87 (0.44–1.72)	—	0.81 (0.41–1.59)	—	0.91 (0.46–1.80)	0.75 (0.40–1.41)
Sex									
Female	73 (57.5)	52 (40.9)	2 (1.6)	0.36		0.25	0.54	0.17	0.37
Male	20 (69)	8 (27.6)	1 (3.5)	0.56 (0.23–1.37)	1.82 (0.16–21.17)	0.61 (0.26–1.44)	2.23 (0.20–25.49)	0.55 (0.23–1.34)	0.70 (0.31–1.56)
Tumor size									
<1 cm	24 (80)	6 (20)	0 (0)	**0.043**		**0.016**	0.33	**0.025**	**0.013**
≥1 cm	63 (56.8)	46 (41.4)	2 (1.8)	2.92 (1.10–7.72)	—	3.05 (1.16–8.04)	—	2.83 (1.07–7.48)	3.03 (1.17–7.84)
N stage									
N0	51 (56)	38 (41.8)	2 (2.2)	0.43		0.77	0.2	0.96	0.61
N1	27 (58.7)	19 (41.3)	0 (0)	0.94 (0.46–1.94)	—	0.90 (0.44–1.84)	—	0.98 (0.48–2.02)	0.84 (0.42–1.66)
TNM stage									
I–II	65 (61.3)	40 (37.7)	1 (0.9)	0.68		0.90	0.41	0.73	0.92
III–IV	20 (62.5)	11 (34.4)	1 (3.1)	0.89 (0.39–2.06)	3.25 (0.19–54.35)	0.95 (0.42–2.15)	3.39 (0.21–55.74)	0.86 (0.38–1.98)	1.04 (0.49–2.22)
Extrathyroidal expansion									
Negative	77 (64.2)	42 (35)	1 (0.8)	0.34		0.38	0.19	0.62	0.24
Positive	9 (52.9)	7 (41.2)	1 (5.9)	1.43 (0.50–4.10)	8.56 (0.49–148.88)	1.59 (0.57–4.43)	7.44 (0.44–124.85)	1.30 (0.46–3.66)	1.77 (0.70–4.48)
Vascular invasion									
Negative	75 (63.6)	42 (35.6)	1 (0.8)	0.49		0.76	0.23	0.96	0.54
Positive	12 (60)	7 (35)	1 (5)	1.04 (0.38–2.85)	6.25 (0.37–106.77)	1.16 (0.44–3.07)	6.16 (0.37–102.69)	0.97 (0.36–2.63)	1.32 (0.54–3.22)
Capsular invasion									
Negative	71 (61.2)	43 (37.1)	2 (1.7)	0.70		0.83	0.40	0.95	0.73
Positive	14 (63.6)	8 (36.4)	0 (0)	0.94 (0.37–2.43)	—	0.90 (0.35–2.3)	*—*	0.97 (0.38–2.50)	0.85 (0.35–2.10)

*Note*: Logistic regression analysis was used for statistical analysis.

Bold values are statistically significant (*p* < 0.05).

No relationship was found between *CTSB* rs12898 G/A polymorphism and clinical findings of PTC (Table 8).

**TABLE 8:** Association of *CTSB* rs12898 G/A polymorphism with clinical characteristics of papillary thyroid carcinoma.

Characteristics	GG	GA	AA	*p*‐value/OR (95% CI)					
				Codominant1	Codominant2	Dominant	Recessive	Overdominant	Log‐additive
Age, years									
<40	41 (40.2)	41 (40.2)	20 (19.6)	0.96		0.77	0.87	0.87	0.78
≥40	23 (42.6)	21 (38.9)	10 (18.5)	0.91 (0.44–1.90)	0.89 (0.36–2.23)	0.91 (0.46–1.77)	0.93 (0.40–2.16)	0.95 (0.48–1.86)	0.94 (0.60–1.46)
Sex									
Female	51 (402.)	52 (40.9)	24 (18.9)	0.81		0.65	0.83	0.52	0.85
Male	13 (44.8)	10 (34.5)	6 (20.7)	0.75 (0.30–1.88)	0.98 (0.33–2.89)	0.83 (0.37–1.86)	1.12 (0.41–3.05)	0.76 (0.33–1.76)	0.95 (0.55–1.64)
Tumor size									
<1 cm	11 (36.7)	15 (50)	4 (13.3)	0.43		0.64	0.35	0.23	0.86
≥1 cm	46 (41.4)	42 (37.8)	23 (20.7)	0.67 (0.28–1.62)	1.38 (0.39–4.79)	0.82 (0.36–1.88)	1.70 (0.54–5.3)	0.61 (0.27–1.37)	1.05 (0.61–1.81)
N stage									
N0	36 (39.6)	39 (42.9)	16 (17.6)	0.99		0.96	0.98	0.94	0.99
N1	18 (39.1)	20 (43.5)	8 (17.4)	1.03 (0.47–2.24)	1.00 (0.36–2.77)	1.02 (0.49–2.10)	0.99 (0.39–2.51)	1.03 (0.50–2.10)	1.00 (0.61–1.64)
TNM stage									
I–II	47 (44.3)	42 (39.6)	17 (16)	0.47		0.95	0.26	0.39	0.53
III–IV	14 (43.8)	10 (31.2)	8 (25)	0.80 (0.32–1.99)	1.58 (0.56–4.43)	1.02 (0.46–2.27)	1.75 (0.67–4.53)	0.69 (0.30–1.61)	0.84 (0.42–1.66)
Extrathyroidal expansion									
Negative	51 (42.5)	45 (37.5)	24 (20)	0.95		0.92	0.82	0.77	0.96
Positive	7 (41.2)	7 (41.2)	3 (17.6)	1.13 (0.37–3.48)	0.91 (0.22–3.83)	1.06 (0.38–2.96)	0.86 (0.23–3.22)	1.17 (0.41–3.28)	0.98 (0.50–1.93)
Vascular invasion									
Negative	50 (42.4)	45 (38.1)	23 (19.5)	0.86		0.84	0.58	0.79	0.67
Positive	8 (40)	7 (35)	5 (25)	0.97 (0.33–2.90)	1.36 (0.40–4.61)	0.10 (0.42–2.90)	1.38 (0.45–4.18)	0.87 (0.32–2.35)	1.14 (0.62–2.12)
Capsular invasion									
Negative	49 (42.2)	44 (37.9)	23 (19.8)	0.71		0.91	0.48	0.51	0.78
Positive	9 (40.9)	10 (45.5)	3 (13.6)	1.24 (0.46–3.32)	0.71 (0.18–2.87)	1.06 (0.42–2.67)	0.64 (0.17–2.34)	1.36 (0.54–3.42)	0.92 (0.49–1.70)

*Note*: Logistic regression analysis was used for statistical analysis.

*p* < 0.05 was considered statistically significant.

## 4 DISCUSSION

PTC is a disorder resulting from the malignancy in thyroid parenchymal cells. This cancer is the most prevalent kind of thyroid cancer, accounting for 80% of all thyroid cancer patients.^6^ PTC is more frequent in the third to fifth decades of life, and the mean age of this cancer is 40 years. Its frequency enhances with age, and is more in women compared with men, in ratios of 2:1 to 4:1.^23^ Despite numerous studies, the exact etiology of this disease is not clear. However, various environmental and genetic factors are known as disease risk factors. Evidence showed that the family history of PTC could consider as an essential factor in PTC predisposition. Therefore, numerous studies attempted to evaluate the association between various genetic variants and PTC.^7^, ^24^

Axis inhibition protein 1 (Axin1) is a scaffold protein with several domains in the β‐catenin destruction complex. It exerts its action via the Wnt/β‐catenin signaling pathway and acts as a negative regulator of this pathway, and can induce apoptosis.^15^, ^25^ Wnt signaling is controlled by the degradation of the Axin1‐mediated β‐catenin destruction complex. AXIN and APC proteins act in the assembly of the β‐catenin destruction complex. The AXIN–APC and AXIN–β‐catenin interactions can contribute to the initiation of β‐catenin phosphorylation and degradation. AXIN1 serves as the main regulator in embryonic development and controls cell proliferation, polarity, and differentiation as well as homeostasis in adult tissue. Therefore, this protein acts as a tumor suppressor, and its defect has been shown to play a central role in carcinogenesis. Lower expression of AXIN1 has been reported in several cancerous cells like hepatocellular carcinoma.^26^ Evidence showed the effect of several genetic variants, including catenin (cadherin‐associated protein), beta 1, AXIN1, and APC, and chromosomal abnormalities in the molecular pathogenesis of Anaplastic thyroid cancer.^23^ Considering the role of AXIN1 protein in tumorigenesis, several studies evaluated the possible roles of genetic variants of the *AXIN1* gene in several cancers.

In the presents study, we assessed the effects of *AXIN1* rs12921862 C/A, rs1805105 G/A and, *CTSB* rs12898 G/A polymorphisms on PTC onset and its clinical findings. This research showed the association between *AXIN1* rs12921862 C/A polymorphism and an increased risk of PTC in all genetic models, except the overdominant model. The frequency of *AXIN1* rs1805105 GA genotype was higher in PTC patients, and also rs1805105 G/A polymorphism was associated an increased PTC risk only in codominant and overdominant models. The frequency of *AXIN1 A*_*rs12921862*_ *A*_*rs1805105*_ haplotype was higher in PTC group and was associated with an increased risk of the PTC. Moreover, the *AXIN1* rs12921862 C/A polymorphism was not associated with PTC findings, but *AXIN1* rs1805105 G/A polymorphism was associated with almost three folds of larger tumor size (≥1 cm).

Although no published report is published on the relation between *AXIN1* polymorphisms and PTC, there are several studies on the association between these variants and various cancers.

In their study, Li et al demonstrated that the *AXIN1* rs12921862, rs1805105, and rs370681 polymorphisms were associated with a higher risk of bladder cancer.^18^ Zhang et al indicated the effects of rs12921862 AC and the rs1805105 GA genotypes on epithelial ovarian cancer (EOC) susceptibility. In addition, they showed that EOC patients with rs12921862 CC genotype improved by the Kaplan–Meier survival curves for the overall survival.^27^ In a meta‐analysis performed on eight studies on the association between *AXIN2* rs2240308 polymorphism and overall cancer risk (2015), Gong et al showed decreased cancer risk in the homozygous, heterozygous, dominant, and allelic (T vs. C) models.^28^ Similar to the finding of the present study, Pu et al showed an increased risk of cell renal cell carcinoma (ccRCC) in individuals carrying rs1805105 GA/GG genotypes and rs12921862 AA genotype. Indeed, the presence of the rs1805105 GA genotype is associated with a 1.92‐fold higher risk of developing clinical stage III and IV cancer.^29^

There was a relationship between *AXIN1* rs9921222 polymorphism and colorectal cancer in the Rosales‐Reynoso study. Despite our results, they showed no association between rs1805105 G/A polymorphism and colorectal cancer. In addition, there was a relationship between advanced Tumor‐Node‐Metastasis (TNM) stages with rs9921222 and rs1805105 G/A polymorphisms and tumor location with rs9921222 variant.^30^ No association between rs1805105 G/A and rs214252 polymorphisms and non‐small cell lung cancer risk was reported by Xu et al.^31^ Similarly, Wang et al found no relationship between several *AXIN1* polymorphisms and Breast Cancer.^32^

In addition, in the current study, no relationship was found between the *CTSB* rs12898 G/A variant and PTC in all genetic models. Also, the *CTSB* rs12898 G/A was not associated with clinical findings of PTC.

Cathepsin B (Ctsp) is a cysteine protease enzyme involved in protein degradation and processing.^12^ Since Ctsb improves tumor progression and its expression is higher in many tumors, it is considered as a cancer biomarker. Evidence revealed that overexpression of *CTSB* could initiate cell invasion and metastasis in several cancers.^13^ Despite the results of the present study, Cui et al indicated the relationship between *CTSB* rs12898 G/A polymorphism and Primary Hepatic Cancer (PHC). They showed a higher risk of PHC in the GA and AA genotypes compared to the AA genotype, and the increased risk of PHC in dominant, recessive and allelic models.^20^ In Stiblar‐Martincic et al's study, a leucine to valine substitution (L26V) polymorphism in the Cathepsin B was indicated in relation to risk of prostate adenocarcinoma. They showed that the VV genotype of this variant be related to higher prostate adenocarcinoma, and less differentiated cancer.^21^ Chen et al showed no association between *CTSB* rs12338, 13,332 and rs8898 variants and oral cancer.^33^ In another study, Chen et al found a significant effect of rs13332 but not 13,332 and rs8898 polymorphisms and hepatocellular carcinoma. Indeed, they showed higher tumor size development in patients carrying the rs12338 variant.^34^ Ma et al study revealed the relationship between rs9009, rs6731, and rs17814426 polymorphisms and lower risk of gastric cancer.^35^

The present study, for the first time, studied the effects of *AXIN1* and *CTSB* polymorphisms on PTC and its clinical findings; however, it suffers from several limitations, first, the low sample size, especially in subgroup analysis which could be significant in a higher sample size. Second, the results would have been more valuable if the study had been performed on cancerous tissue and healthy lateral tissue.

In conclusion, our findings suggested that the *AXIN1* rs12921862 C/A polymorphism was associated with an increased risk of PTC in all genetic models, except the overdominant model. The *AXIN1 rs1805105 G/A* polymorphism was associated with an increased PTC risk only in codominant and overdominant models. The *AXIN1 A*_*rs12921862*_ *A*_*rs1805105*_ haplotype was associated with an incresead risk of PTC. The *AXIN1 rs1805105 G/A* polymorphism was associated with almost three folds of larger tumor size (≥1 cm). The CTSB rs12898 G/A variant showed no effect on PTC and its clinical findings.

## CONFLICT OF INTEREST

All the authors declare no conflict of interest to disclose.

## Data Availability

The data are available from the corresponding author on reasonable request.
